# Mechanical Properties of Endothelial Cells: A Key to Physiology, Drug Testing and Nanostructure Interaction

**DOI:** 10.3390/cells14211659

**Published:** 2025-10-23

**Authors:** Agnieszka Maria Kołodziejczyk, Łukasz Kołodziejczyk, Bolesław Karwowski

**Affiliations:** 1 Food Science Department, Faculty of Pharmacy, Medical University of Lodz, Muszyńskiego 1, 90-151 Lodz, Poland; agnieszka.kolodziejczyk@umed.lodz.pl (A.M.K.); boleslaw.karwowski@umed.lodz.pl (B.K.); 2 Institute of Materials Science and Engineering, Lodz University of Technology, Stefanowskiego 1/15, 90-537 Lodz, Poland

**Keywords:** atomic force spectroscopy, nanoindentation with an AFM tip, endothelial cells, elasticity parameter, nanostructures, finite element method

## Abstract

**Highlights:**

**What are the main findings?**

- Endothelial elasticity is a physical parameter that describes physiological changes in cells.
- Changes in endothelial cell mechanical properties are largely associated with cellular cytoskeleton remodeling.

**What are the implication of the main finding?**

- Force spectroscopy is a relevant method for testing drugs on the endothelium.
- Nanostructures affect the mechanical properties of endothelial cells.

**Abstract:**

This article explores the application of atomic force spectroscopy in in vitro studies of endothelial cells. In this technique, derived from the atomic force microscopy, the AFM probe is employed as a nanoindenter. This review aims to discuss the nanomechanical properties of endothelial cells alongside selected biological parameters used to determine their physiological state. Changes in cellular elasticity are analyzed in the context of an intracellular mechanism involving nitric oxide, prostacyclin, calcium ions and reactive oxygen species levels. The manuscript compiles various articles on endothelial cells, assessing the impact of different agents such as drugs, cytokines and nanostructures. The review article addresses the endothelial dysfunction model, which is based on alteration in the mechanical properties of the cells, and explains how this model is used for potential drug testing. The next part of the study evaluates the toxic effects of nanostructures on endothelial cells. Additionally, the article addresses the finite element method, a promising new approach for modeling and simulating the behavior of cells treated as a multi-layered structure.

## 1. Introduction

A cell is the smallest building block of human body that affects its entire functioning. Cells are most often analyzed in terms of their biological, biochemical and physical parameters. One of the physical parameters is elasticity, i.e., the ability of a material to return to its original shape after removing external forces. Elasticity depends on particular functions and location of cells [1,2,3]. This review discusses the most relevant articles related to the study of the mechanical properties of endothelial cells (ECs). The endothelium is the layer of cells lining the blood vessels, and its cells can be defined as microsponges that interact both with each other and the surrounding environment [4]. It mediates the transport of many factors from the blood to deeper tissues, thus regulating the functioning of the human body [5,6]. The sensation of external stimuli, including external forces, can activate membrane proteins submerged in the lipid bilayer to trigger the intracellular physiological or pathological action [7,8,9]. Blood vessels are subjected to constant compression and stretching, hence the elastic properties of endothelial cells become an important parameter reflecting its proper functioning [10]. The elasticity of the endothelium can also alter due to atherosclerotic plaque settled on its surface [11]. For the physiological state of the endothelial cell, the native values of its elasticity have been determined [12].

**Figure 1:**
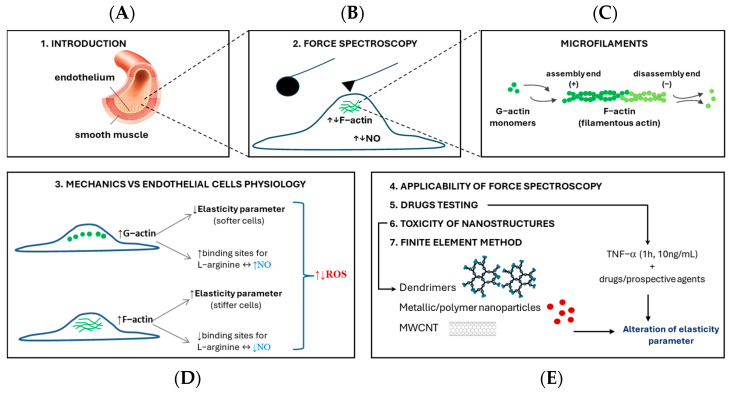
Schematic representation of the manuscript structure. First, the location of endothelial cells in the human body is presented (**A**). Section 2 refers to force spectroscopy methodology (**B**), followed by a detailed description of microfilaments, which are connected to the polymerization of F-actine (decrease in G-actin content) (**C**). Section 3 discusses the relationship between cell stiffness and selected physiological parameters including, e.g., nitric oxide (NO), and reactive oxygen species (ROS) formation (**D**). Section 4, Section 5, Section 6 and Section 7 are depicted schematically (**E**). Section 4 introduces the applicability of force spectroscopy in in vitro endothelial cell studies. The manuscript primarily discusses testing drugs and potential substances on a Tumor Necrosis Factor-α model (1 h, 10 ng/mL) (Section 5) and assessing the impact of nanostructures on endothelial cell elasticity (Section 6). The comparative analysis of the influence of nanostructures concerns metallic and polymer nanoparticles, dendrimers, and multi-walled carbon nanotubes (MWCNT).

This review compares the most relevant findings obtained using atomic force spectroscopy (AFS) for endothelial cells. The originality of this approach is associated with a pioneering study on the application of the elasticity parameter as physiological relevance marker. Figure 1 shows the structure of the manuscript, including its individual sections.

In order to accurately interpret the results of cell elasticity and demonstrate their physiological meaning, selected biological methods are employed for insightful analysis. These frequently used methods are labeling of the cytoskeleton with fluorescence imaging, measurements of the levels of biological markers, such as nitric oxide, prostacyclin, intracellular calcium, or labeling of reactive oxygen species production. Methodological aspects of elasticity parameter measurements (Section 2), endothelial cell physiology and nanomechanics (Section 3) have been further presented. The applicability of force spectroscopy measurements were presented in Section 4. Finally, the model of endothelial dysfunction used to test drugs and potential endothelial factors was discussed (Section 5). Additionally, this review includes an in-depth analysis of the elasticity of cells exposed to nanostructures (Section 6). The finite element method section highlights works in which the authors developed a force feedback simulator for AFM indentation analysis and relaxation tests (Section 7).

## 2. Different Approaches to Force Spectroscopy Measurements and Analysis

### 2.1. Introduction to Force Spectroscopy Technique

**Figure 2:**
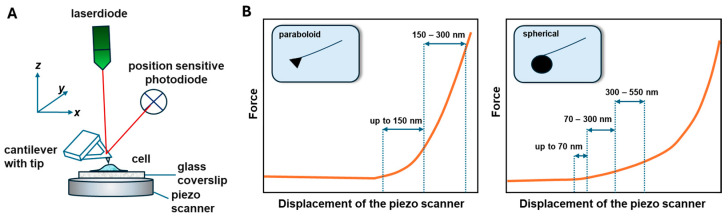
The scheme of an atomic force microscope (AFM) (**A**), and approach force–distance curves for a paraboloid and a spherical probe (**B**). Figure (**B**) adapted with permission from Ref. [20]. 2021, Elsevier. The depths of the indentations and contact point have been marked for illustrative purposes.

Atomic force spectroscopy (AFS) is a method that enables measurement of cell elasticity [13,14,15]. It is a nanoindentation technique that uses a probe of an atomic force microscope. Briefly, the cell is pressed with a certain force using the AFM, and then the so-called approach force—distance curve (force in the function of the displacement of piezoelectric scanner) is recorded. The study of cell elasticity using force spectroscopy involves an analysis of the penetration of an AFM probe into the tested material as a function of force [16]. Subsequently, an AFM probe is removed from the cell surface, and a retract curve is collected. Native (living) cells have a very delicate structure; therefore, the applied forces used to measure force–distance curves are approximately 1 nN [17,18]. It is also very important to select suitable AFM probes in terms of the radius of curvature and the spring constant of the cantilever. For AFS measurements of soft materials, probes with low cantilever stiffness are preferred [18]. Atomic force microscopes enable the measurement of force–distance curves at any point in the image, measurement in grids of points or mapping mechanical properties within both cell fragments and its entire area. Figure 2 shows a scheme of an atomic force microscope (AFM) (A) and force–distance for paraboloid and spherical probes (B). In AFM imaging mode, the image is recorded based on the displacement of the reflected laser beam on a quadrupole detector (Figure 2A). The position of the laser spot on the photodiode depends on the deflection of the cantilever (with the AFM tip) onto which the laser beam falls. AFM imaging allows registration of the topography of cell surface (Figure 2A), in particular precise localization of the cell nucleus, where force–distance curves (schematically depicted in Figure 2B) are most often collected in spectroscopy mode of AFM. Analysis of the force–distance curves of piezoelectric scanner enables calculation of the cell elasticity, known as the cell elasticity parameter [17,19,20,21]. To this end, an indentation curve is used, representing the difference between approach curves collected on the tested cell and the reference sample (treated as a rigid, non-deformable material such as glass).

The elasticity parameter is determined experimentally by fitting the curve in the Hertz [22] or Sneddon [23] models (the most frequently chosen analysis model). In many articles, cell elasticity values are calculated as Young’s modulus [24], apparent Young’s modulus [25], or the modulus or elasticity parameter. In the case of cell elasticity measurements, the value of an elasticity parameter determined experimentally depends mostly on current cell deformation. Formally, however, the modulus of elasticity should characterize the material regardless of deformation within the elastic strain regime (Hooke’s law). Therefore, the term ‘elasticity parameter’ seems most appropriate. Guz et al. suggested that Young’s modulus be redefined at the nanoscale [26]. It should be noted that different terminology is used in the literature. Authors define the calculated value (e.g., elasticity parameter or stiffness) and indicate which model was used for the calculations.

### 2.2. Comparison of Force Spectroscopy Study with Different Probes Geometries and Materials

The quality and reliability of biophysical measurements acquired by Atomic Force Microscopy (AFM) are highly reliant on the AFM probe’s properties. The shape, material characteristics, and surface chemistry of the probe [26], which serves as the principal interface between the instrument and the biological sample, can have a substantial impact on the mechanical response recorded during indentation studies, as noted by Okajima [27]. Nanometer-size probes in the shape of a paraboloid or a cone can be used, and the elasticity measured with these probes is considered a local response [18]. The shape of the probe determines the model used for force–displacement curve analysis. Another option is to measure the AFS using a spherical probe in a size appropriate for a micrometer, through which the global response is collected [28,29,30]. Spherical colloidal probes distribute force across a greater contact surface, lowering stress and supporting more accurate modeling using Hertzian or Sneddonian contact mechanics, as demonstrated by Kulkarni et al. [31] and Brill-Karniely [32]. Regardless of the size of probe selected, the indentation depth should be less than 10% of the cell height to minimize the effects of the stiff substrate (e.g., glass) on which the cells are cultured [20]. Other investigations [33,34] have emphasized the depth of indentation and the rate at which force is applied as key parameters influencing the reported mechanical response. Shallow indentations primarily test the cell membrane and cortical cytoskeleton, but deeper indentations contact intracellular components and may transfer force to the substrate. Similarly, fast loading rates highlight elastic behavior, but slower rates allow viscoelastic features to develop. AFS method can still be used for cultivation on soft materials, including those with a defined modulus of elasticity, as detailed in the sections on the substrate influence and limitations of using AFS (see Section 2.3. Substrate influence on force spectroscopy measurements and Section 2.5. Limitation of force spectroscopy study). In the case of a nanometer-size probe, the cortical part of the cell cytoskeleton located just below the cell membrane is more sensible. With a micrometer spherical probe, it is possible to detect cell elasticity from the glycocalyx, cellular membrane, cytoskeleton, cytoplasm with organelles and the cell nucleus. In our previous article [20] a detailed analysis of force—distance curves at the cellular level using a sharp, spherical probe has been presented. This study involved analyzing endothelial cell elasticity using sharp probe measurements across two indentation ranges, i.e., up to 150 nm and from 150 to 300 nm. Thus, cell elasticity changes in the cell membrane and cortical cytoskeleton could be detected in the first range of indentations, and changes in elasticity in the deeper cytoskeleton, tubulins, stress fibers and cell organelles located at indentation depths of up to 300 nm were identified in the second range of analysis. The elasticity parameter values measured with paraboloid probe are more than approx. 2–3 times higher that obtained with spherical probe [35,36,37,38]. Data analysis at different cellular levels aims to detect changes in cell elasticity resulting from changes in the cell phenotype. It also allows for studying the location of nanostructures inside cells [20]. An important advantage of elasticity measurements using a micrometric spherical probe is the ability to detect changes in glycocalyx stiffness [39,40,41], which can lead to various pathological changes in endothelial cells. Conversely, nanometer-size probes are preferable for mapping mechanical properties.

The mechanical characteristics of the probe material, which is usually silicon or silicon nitride, affect its stiffness and sensitivity. Furthermore, the surface chemistry of the probe can influence cell-probe interactions [42]. Hydrophobic or charged surfaces may disrupt membrane adherence or cause unexpected biochemical reactions. As shown in the work of Variola [43] and Toca-Herrera [44], functionalization of the probe with biomolecules (e.g., ligands, antibodies) is often used in force spectroscopy to examine particular receptor-ligand interactions.

### 2.3. Substrate Influence on Force Spectroscopy Measurements

The mechanical properties of adherent cells evaluated with AFM are strongly affected by the mechanical properties of the underlying substrate. Jaddivada and Gundiah [45] found that it is especially important when cells are thin or soft, since the indentation force produced by the AFM probe can propagate into the cell and deform the substrate, resulting in falsely exaggerated stiffness values. When a cell is cultured on a stiff substrate like glass or plastic, the AFM probe may compress both the cell and the substrate during indentation. This produces a composite response that incorporates contributions from both the cell and the substrate. Cells on compliant substrates (e.g., hydrogels) deform more locally, allowing for more precise determination of intrinsic cellular mechanics, as demonstrated by Califano and Reinhart-King [46]. To overcome this, researchers frequently adopt corrective models, such as the composite Hertz model, which takes into consideration the layered architecture of the cell–substrate system. Alternatively, as established by Ding et al. [47] and Vichare et al. [48], finite element modeling (FEM) can mimic the indentation process and distinguish between the contributions of the cell and substrate. The substrate effect is also affected by the cell’s height and form. For example, in thin areas of the cell (such as lamellipodia), the probe may be able to reach the substrate more readily, leading to the mechanical output. On the other hand, measurements made over the nucleus or thicker cytoplasmic areas are less impacted by substrate deformation. Beyond measurement inaccuracies, substrate stiffness can influence cellular function via mechanotransduction pathways. Cells detect and respond to substrate mechanics through focal adhesions, which connect the extracellular matrix and the cytoskeleton via integrins. Changes in substrate stiffness may modify cytoskeletal tension, adhesion dynamics, and intracellular signaling, affecting the cell’s mechanical characteristics [45]. For example, cyclic stretch on compliant substrates has been found to enhance cytosolic calcium levels, integrin recruitment, and traction forces, but excessive stiffness can destabilize focal adhesions and cause cell deadhesion [45].

Rheinlaender et al. [49] studied the link between substrate stiffness and cortical cell dynamics using atomic force microscopy. Interestingly, contrary to the generally held idea that substrate stiffness has a substantial impact on observed cell stiffness, the study found that cortical stiffness of cells is mainly independent of the mechanical characteristics of the substrate. This study raises issues with the concept that substrate effects invariably corrupt AFM-based stiffness measurements, implying that, under specific conditions and measurement techniques, intrinsic cellular mechanics can be successfully measured with minimal substrate interference.

To reduce substrate influence in AFM experiments, several strategies are used: lowering the indentation depth helps avoid probe interaction with the substrate; compliant substrates are selected to better replicate physiological conditions; correction models or finite element methods (FEM) are applied to interpret force-indentation data more accurately; and measurements are performed over thicker regions of the cell, such as the nucleus, where substrate effects are not as significant. These aspects are critical for accurately interpreting AFM-based mechanical results and understanding cells’ real viscoelastic behavior.

### 2.4. Elasticity and Viscoelasticity of Cells

Cellular elasticity is a capacity of cell to withstand deformation when subjected to an applied force. In AFM, this is commonly measured using force–distance curves, which plot the probe’s indentation depth into the cell versus the applied force. The Young’s modulus is calculated from these curves using models such as the Hertz or Sneddon models, which vary based on the geometry of the probe and the nature of the sample, as described by Cappella [50]. The Hertz and Sneddon models are commonly used to evaluate force-indentation curves and determine the elasticity modulus. However, these models are based on idealized assumptions, such as completely elastic, homogeneous, isotropic, and semi-infinite materials, which are rarely observed in biological systems. The Hertz model, which assumes spherical indentation, does not account for adhesion forces or substrate effects, whereas the Sneddon model, formulated for conical indenters, assumes perfect tip geometry and linear elasticity [51]. In practice, AFM tips frequently deviate from ideal forms resulting from wear or manufacturing variation, and cells react nonlinearly to mechanical stress. Both models are extremely susceptible to contact point determination mistakes, which can result in large errors in modulus determination [52]. Additionally, neither model takes into consideration time-dependent mechanical responses, which are essential for interpreting cellular activity in physiological and pathological conditions [53]. Recent research has shown that matrix stiffness, cytoskeletal remodeling, and pharmacological treatments can significantly modify cellular elasticity, further complicating the use of classical models [54,55].

Beyond purely elastic behavior, cells also exhibit viscoelastic behavior, which means that their mechanical response contains both elastic (instantaneous) and viscous (time-dependent) components caused by their high water content and polymeric structural matrix. Cellular components such as the cell membrane, cytoskeleton, and cytoplasmic viscosity all have an impact on cell viscoelasticity. Studies by Fletcher and Mullins [56], Pegoraro et al. [57], and Trickey et al. [58] indicate the cytoskeleton, which includes microtubules, intermediate filaments, and microfilaments, as a key contribution in cell viscoelasticity. AFM can capture this through carrying out force-relaxation or creep experiments, which involve holding the probe at a constant indentation and recording the force decline over time. This approach has been effectively demonstrated by Chim et al. [59], and further explored in broader contexts by Mba et al. [60] and Bishawi et al. [61]. Alternatively, dynamic mechanical analysis with oscillating load can reveal frequency-dependent mechanical features, which are typically expressed using power-law rheology, as discussed by Okajima [27].

Recent developments allow AFM to quantify these characteristics in near-physiological conditions, leading to more precise characterization of cellular mechanics essential to biological function and pathological states, as emphasized by Alsteens [62]. Although force–displacement curves are routinely employed in AFM to assess the local, nanoscale elastic characteristics of soft materials, a theoretical foundation for obtaining viscoelastic constitutive parameters from force–displacement data is still not provided [63]. Recent advances have verified a strategy based on the elastic–viscoelastic correlation principle using finite element modeling (FEM) and comparisons to current in vivo cell and hydrogel AFM techniques.

### 2.5. Limitation of Force Spectroscopy Study

For AFS measurements, cells are usually cultured on a rigid substrate, such as a coverslip or slide. However, force spectroscopy studies do not exclude other substrates for cell culture [64,65], and it is also possible to culture cells on soft substrates, which is important in the case of endothelial cells. In particular, it is possible to culture cells on materials with precisely defined elasticity moduli, or on synthetic and naturally derived polymeric hydrogels such as PEG, polyacrylamide, hyaluronic acid, or collagen [64]. Culturing on soft materials brings the research model closer to the physiological one, where endothelial cells are located on soft cells in the human body [66]. The research cited in the manuscript refers to in vitro studies. Endothelial cell culture should be carried out in accordance with the specifications of the particular cell supplier, most often at a temperature of 37 °C and 5% CO_2_. Modern AFM microscopes are equipped with dedicated heating pads that allow physiological temperature to be maintained [67]. Furthermore, it is also possible to conduct research using a so-called perfusion cell, which allows, for example, the medium to be changed during the test (from the reference medium to a medium containing a drug or nanoparticle colloid) and thus to monitor changes in survivability [68]. The limitations of force spectroscopy are primarily related to the difficulty or even impossibility of testing cells in suspension. The material for indentation should be tightly bound to the substrate, i.e., it should not move during the analysis of elastic properties. From a technological perspective, many microscopes are designed with an additional optical view from below, which causes limitations in the case of non-transparent samples.

Artifacts that involve probe-induced deformation, adhesion, and lateral slippage can distort force–distance curves and impair data interpretation [69]. Alsteens [70] and Abu Qubaa et al. [62] observed that high adhesion between the probe and the cell surface might cause hysteresis or changed retraction profiles, whereas lateral movement during indentation can add shear forces that are not accounted for in typical elastic models. To limit these effects, researchers use techniques such as surface passivation to reduce probe-cell adhesion, low-indentation depths, and correction algorithms during data processing.

In the present manuscript, only the limitations of force spectroscopy are highlighted, but this does not fully cover the various technological problems that product engineers and scientists are trying to overcome.

## 3. How Changes in the Mechanical Properties of Cells Affect Their Physiology

In the article by Cines et al. [71], the endothelium is described as a dynamic heterogeneous, disseminated organ that has vital secretory, synthetic, metabolic, and immunologic functions. It is worth mentioning that heterogeneity of endothelial cells may contribute to the maintenance of adaptive processes [72]. On the other hand, the dynamic heterogenous nature of the endothelium may lead to the development of disorders limited to specific vascular beds [73]. One of the roles of endothelial cells is the formation of new blood vessels (angiogenesis). Additionally, they constitute a matrix for the proper movement of various macro- and microparticles, gases and fluids from the blood to tissues. This movement also occurs in the opposite direction, i.e., as secretion of various essential substances into the blood. The two-way movement of these components is associated with the control of flowing blood pressure. The main endothelium-derived relaxing factors are nitric oxide (NO) and prostacyclin (PGI_2_), whose principal role is vasodilation [74], whereas vasoconstriction requires the (a) platelet-activating factor (PAF) and (b) endothelin-1 (ET-1), synthesized by the endothelium. Nitric oxide inhibits platelet and leukocyte activation and maintains the vascular smooth muscle in a non-proliferative state [75]. Understanding the state of the inflamed endothelium constitutes an important issue in the context of cardiovascular diseases. Abnormalities of endothelial cells may be associated with altered cell structure, disturbed physiology, manifested as an increase or decrease in vasoactive factors that may also change the nanomechanical properties of the cells [76].

### 3.1. Mechanical Environment of the Endothelium

Sources of mechanical stress on the endothelium can be divided into two categories: contact stress, which results from the physical characteristics of the substrate, such as topography [77,78], curvature [79], stiffness [80,81]; and stresses resulting from blood flow (shear stresses) [82,83], tensile stresses [84,85] and hydrostatic pressure [86,87]. These issues are described in detail in the article of Dessalles et al. [88]. Stresses originating from fluid consist of shear stress on the apical surface of endothelial cells caused by viscous blood flow, compressive blood pressure, and circumferential and axial tensile stresses caused by transmural pressure differences and tissue movement, respectively. Furthermore, the presence of blood cells can significantly modify the flow dynamics near the vessel wall, which in turn impacts the shear stress that endothelial cells experience [89]. These mechanical signals in vivo act upon endothelial cells and indirectly influence the deeper cells within the blood vessel lumen. Endothelial cells interpret and integrate these mechanical stimuli. While the physical characteristics of the substrate may stay constant over brief periods, shear, compressive, and tensile forces are subject to significant fluctuations and change cyclically due to the pulsation of blood flow [90]. Additionally, the type and intensity of these mechanical signals differ based on their location within the vascular system and various organs. For instance, in vivo, the time-averaged wall shear stress is approximately 1 Pa in the aorta, 5 Pa in small arteries, 2 Pa in venules and 0.1 Pa in the vena cava [91]. Mechanical stimuli affect the morphology, intracellular organization and functions of endothelial cells. Conversely, endothelial elasticity is an important parameter that describes proper functioning.

### 3.2. Elasticity of Endothelial Cells

Interestingly, the elasticity of endothelial cells is primarily associated with the level of nitric oxide secreted by the endothelium and polymerization/depolymerization processes of cellular cytoskeletal actin [17,19,30,41]. The cytoskeleton of endothelial cells consists of a network of protein fibers, including microfilaments, microtubules and intermediate filaments. Groups of Rotsch and Radmacher [92] were the first to investigate that cell mechanics depend primarily on actin filaments, whereas microtubules play a minor role. Measured elasticity is mostly affected by microfilaments concentrated mainly in the cortex layer, just below the cell membrane. These structures are composed of two forms of actin, i.e., G-actin (globular, monomeric) and F-actin (fibrillar) [93]. They are linked to each other through polymerization and depolymerization processes. A single F-actin filament is a polarized structure with a diameter of about 7 nm. G-actin monomers attach to the positive end and detach from the negative end. The formation of F-actin is associated with creating of the so-called “enucleation sites” composed of three G-actin monomers to which the profilin protein is attached. The detachment of profilin induces the polymerization of actin fibers, forming fibrillar actin. It is important to note that fibrillar actin can form bundles of actin filaments called “stress fibers”. These fibers are anchored to the cell membrane by their positive ends and are typically arranged parallel to the stress line of the cytoplasm [94]. F-actin is a component of the cytoskeleton responsible for the shape and elastic properties of cells. A higher level of F-actin reduces cell elasticity. It is also associated with cell migration and the regulation of endothelial monolayer permeability [95]. On the other hand, a higher level of G-actin in cells (depolymerization of F-actin) results in an increase in binding sites for eNOS synthases and enhanced substrate (L-arginine) transport for NO synthesis [96]. L-arginine enters endothelial cells primarily via cationic amino acid transporters (CATs), especially CAT-1 [97]. These transporters are anchored to the plasma membrane, and their positioning and mobility are regulated by actin filaments. Thus, disruption of the actin cytoskeleton (e.g., with cytochalasin D) can reduce transporter activity, most probably by altering membrane microdomains or trafficking. Additionally, monomeric G-actin is the main component of the 51 kDa ribonucleoprotein that binds to the posttranslational region of eNOS mRNA 3′ [98]. Therefore, the organization of cytoskeletal actins (and more precisely, the depolymerization processes that increase the amount of G-actin in cells) leads to higher expression of eNOS. Thus, higher levels of G-actin in cells increase nitric oxide, a vasodilator mediator of the vascular endothelium. As reported in the articles [17,99,100], the cell elasticity parameter anti-correlates with the level of nitric oxide.

**Figure 3:**
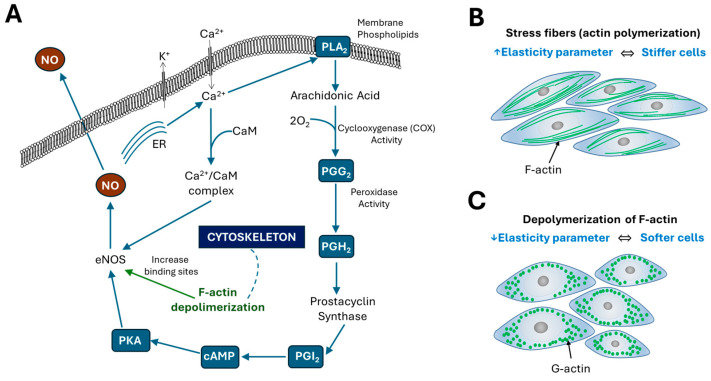
Scheme of the relationship between the PGI_2_ release, nitric oxide synthesis and actin fibers rearrangement of endothelial cells [99,101,102] (**A**) and schematically presented cytoskeleton actin fibers for polymerization (**B**) and depolymerization (**C**) processes along with alteration of elasticity parameter.

Figure 3 presents a scheme of the relation between the elasticity parameters of endothelial cells and biological parameters, such as the levels of nitric oxide, prostacyclin, intracellular calcium ions and reactive oxygen species, in parallel with the polymerization/depolymerization processes of cell actin.

Various external factors, including cytokines, hormones, drugs, nanostructures, etc., can increase the amount of calcium ions in cells. There are two pathways leading to a higher level of intracellular calcium, i.e., the opening of calcium-permeable channels or the re-lease of calcium from intracellular organelles (mainly the endoplasmic reticulum, ER) [103,104]. The amount of Ca^2+^ ions flowing in depends on the electrochemical gradient, which is modulated by the membrane potential. Intracellular calcium ions can form a complex with calmodulin (Ca^2+^/CAM complex), which binds to eNOS, inducing NO production [105]. Furthermore, depolymerization of F-actin causes an increase in the number of eNOS binding sites, which results from increased eNOS activity. It also increases NO release [60]. In turn, intracellular calcium ions can activate phospholipase A_2_, an enzyme found in the cell membrane that hydrolyzes membrane phospholipids and releases arachidonic acid (AA), a precursor of many inflammatory and vasoactive mediators [106]. Arachidonic acid is then converted by the COX-1 and COX-2 (cyclooxygenases) enzymes into intermediate compounds, i.e., prostaglandins PGG_2_ and PGH_2_. Subsequently, PGH_2_ is further metabolized by the PGIS (prostacyclin synthase) to prostacyclin (PGI_2_) [107,108,109]. Prostacyclin binds to the prostacyclin receptor (IP receptor), coupled to the Gs protein and located on endothelial cells surface. Activation of the receptor leads to increased concentration of cyclic adenosine monophosphate (cAMP), which results in the activation of protein kinase A (PKA). The protein kinase then phosphorylates the endothelial nitric oxide synthase (eNOS) enzyme, stimulating its activity and intensifying the production of nitric oxide (NO) [110]. Also, excess reactive oxygen species can inactivate nitric oxide by forming peroxynitrite (ONOO^−^), which is cytotoxic [111,112].

Changes in cell elasticity are also accompanied by changes in morphology. For in-stance, when cells elongate, an increase in stiffness and the appearance of stress fibers can be observed [113]. Conversely, softer cells tend to be more spherical in shape [113]. The elasticity of cells, supported by biological parameters, allows for a proper understanding of the underlying nanomechanical processes occurring at the single-cell level.

## 4. The Applicability of Atomic Force Spectroscopy in the Study of Endothelial Cells

The applicability of atomic force microscopy and spectroscopy to cell investigation is very broad [114,115]. One interesting area is the characterization of endothelial glycocalyx (eGlx) in vitro and ex vivo experiments [116,117,118], which conveys mechanical signals triggered by blood flow to cells. This influences vascular tension and intercellular adhesion. An intact endothelial glycocalyx can serve as a protective barrier against infection.

Another important application of force spectroscopy is the study of the dynamic morphology of fenestration of liver sinusoidal endothelial (LSECs) cells [119,120,121]. These fenestrations, which are patent transcellular pores, are necessary for healthy liver function; however, they degenerate in pathological circumstances, including inflammation and liver fibrosis. Zapotoczny et al. [119] traced the dynamic rearrangement of the cell actin cytoskeleton connected with the formation or closing of cell fenestrations within 1 min, as revealed by topographical and nanomechanical data. Interestingly, pathological LSECs from the genetic model of systemic inflammation, which is triggered by the deletion of Mcpip1 in myeloid leukocytes, regained their fenestrations when cultured on soft hydrogels [120]. Another article [122] provides data showing that endothelial miR-34a deletion protect against aneurysm development despite endothelial dysfunction. This is supported by the results of the aortic wall stiffness study at the endothelial layer in wild type (WT) mice [122]. Kubisiak et al. [123] demonstrated that SARS-CoV-2 infection of HPAECs results in a loss of cell elasticity, which correlates with an increased polymerization of actin filaments and induction of the inflammatory response. The authors of the article [123] indicated that nonproductive SARS-CoV-2 infection, associated with loss of the endothelium elasticity, may be clinically relevant and result in tissue dysfunction and damage. The next article [124] identified the viral nonstructural protein 1 (NS1) as a key player in disrupting endothelial integrity and inducing vascular hyperpermeability independently of pro-inflammatory cytokines. These results provide a framework for understanding the mechano-pathology of dengue virus and lay the groundwork for developing targeted therapeutic strategies to mitigate severe disease outcomes in the future [124].

Numerous studies have revealed [125,126,127] the connection between diabetes and endothelial nanomechanics, which may also highlight the applicability of endothelial elasticity in in vitro and ex vivo investigations. For example, Targosz-Korecka et al. [127] showed that stiffening of endothelial cells and diminished glycocalyx coverage occurred in early diabetes, followed by a reduction in glycocalyx length that correlated with the diabetes progression. This was confirmed in ex vivo experiments using a model of C57BLKs/J-db/db diabetic and control mice at aged 11–19 weeks [127]. Wang et al. [128] demonstrated that glucose levels can notably influence the stiffness of human umbilical vein endothelial cells (HUVECs), subsequently leading to significant alterations in cell migration and proliferation capabilities, which are closely related to tissue regeneration.

It should also be noted that force spectroscopy has specific methodological applications in the study of cancer [129]. These include evaluating the elasticity of cancer cells in comparison to healthy cells [130]. Laurent et al. [96] determined the adhesion force between an endothelial cell monolayer and tumor cells with different metastatic potentials. They found that the most invasive cell lines (T24, J82) formed the strongest bonds with endothelial cells [131]. Interestingly, authors [131] have reported that ICAM-1 acts as a key receptor on endothelial cells, and that its interaction with ligands expressed by tumor cells correlates with rupture forces obtained with the most invasive cancer cells (T24, J82). However, endothelial ICAM-1 does not appear to be involved in the adhesion process for less invasive cancer cells (RT112). Additionally, Csonti et al. [132] investigated the adhesive properties of living breast adenocarcinoma cells to brain endothelial cells and pericytes. They found that the adhesion force of tumor cells varies depending on the target cell type, with notably stronger adhesion to pericytes than to endothelial cells. The results from the studies [131,132] highlight the importance of specific mechanical interactions between tumor cells and host cells during metastasis formation. Although investigating cancer using AFS is a very relevant topic, the wide range of this subject means it is beyond the scope of this manuscript.

## 5. From Cytokine to Drugs—How to Test Potential Agents Based on Elasticity Measurements

In the last decade, numerous studies have been conducted on the application of atomic force spectroscopy as a tool, not only for measuring morphology, but also as a mechanical nanosensor for characterization of endothelial cells and, in particular, various processes associated with their physiological or pathological state [20,40,133,134]. Using AFM techniques, Kushe-Vihrog et al. [133] showed that human endothelial cells derived from umbilical vein (HUVEC) increase their volume and surface area after activation of aldosterone, which is associated with increased expression of endothelial sodium channels (ENaC) and disruption of sodium transport. On the other hand, the use of aldosterone antagonists, spironolactone and amiloride, reverses the effect of aldosterone, thus reducing cell surface and number of ENaC channels. Additionally, force spectroscopy can be useful in testing endothelial dysfunction, e.g., to determine the effect of steroid hormones such as estradiol and aldosterone on the volume and elasticity of the cells [135].

Another factor that causes non-physiological changes in endothelial cells and affects their elasticity is the pro-inflammatory cytokine TNF-α [17,19,99]. In our previous studies [17], it has been shown that HMEC and EA.hy926 cells exhibit a two-step response to this cytokine when administered at a concentration of 10 ng/mL for 1, 3, 6, 12 or 24 h. In the case of short incubation times of 1 and 3 h, an increase in cell elasticity (of about 35 and 25%, respectively) is observed, which is related to the formation of stress fibers and a reduction in G-actin and NO production [99]. Conversely, incubating endothelial cells with TNF-α for a longer period (12 and 24 h) cause in a decrease in elasticity (of about 25 and 20%, respectively), which is associated with depolymerisation of actin fibers and excessive NO efflux [17,19,99]. The short-term endothelial pro-inflammatory activation by TNF-α is quickly followed by a second phase when the cell undergoes functional transformation, with a significant drop in cell elasticity parameter, which renders the cells more susceptible to shear stress mediated by blood flow [136]. The latter condition corresponds directly to increased permeability of blood vessels as well as complex pro-inflammatory and pro-thrombotic phenotypic alterations in the endothelium, and it can lead to the persistence of the endothelial pro-inflammatory phenotype [17].

One of the key factors in investigating the desired effects of drugs or potential therapeutic agents is to define an in vitro model of endothelial cell dysfunction. Such a model was defined as administration of TNF-α to endothelial cells at a concentration of 10 ng/mL for one hour, as detailed in articles [19,99,137]. Based on the above mentioned model the effects of simvastatin [137] and three potential drugs, i.e., 1-methylnicotinamide chloride (1-MNA) [99], 1.4-dimethylpyridine chloride (1.4-DMP), and 1-methylpyridinium chloride (1-MP) [137] were analyzed. The protective effect of simvastatin was demonstrated when it was administered to the HMEC cell line at a concentration of 1 µM for two hours [137]. There was no change in cell elasticity or inflammatory reaction after incubation with the TNF-α cytokine. The therapeutic effect of simvastatin administered at a concentration of 1 µM for two hours is related to the reduced elasticity of cells exposed to simvastatin in an inflammatory cell model compared to cells incubated only with a pro-inflammatory cytokine. The results of the elasticity measurements indicate that a concentration of 0.1 µM of simvastatin is insufficient to produce an anti-inflammatory effect [137]. The findings presented in our previous study prove that 1-methylnicotinamide chloride applied at a concentration of 100 nM for three hours reduces the elasticity parameter of cells below the control value in the dysfunctional endothelial cell model [99]. Whereas, extending the incubation time of 1-methylnicotinamide chloride in the dysfunctional endothelial cell model effectively counteracts the increase in elasticity parameter caused by the pro-inflammatory cytokine TNF-α [99]. As for prospective drugs, the value of the cell elasticity parameter is similar to that obtained for reference (untreated) cells when HMEC cells are first incubated with 1-methylpyridinium chloride for three hours, which is followed by exposition to TNF-α for one hour [137]. However, it does not occur in a therapeutic configuration where the compounds, i.e., 1-methylpyridinium chloride and cytokine, are administrated to the cell culture in reverse order (first cytokine and then 1-methylpyridinium chloride). In both configurations of the endothelial cell dysfunction model, 1.4-dimethylpyridine chloride does not exhibit anti-inflammatory activity [137].

The elasticity parameter can also be used to evaluate the impact of anthracycline antibiotics on endothelial cells. The article by Wójcik et al. [138] presents a broad analysis to assess the effect of doxorubicin (DOX) and daunorubicin (DNR) on endothelial cells in the concentration range of 0.1–10 µM. These agents are widely used in chemotherapy, exerting a cytostatic or cytotoxic effect on cancer cells [139]. Endothelial cells exposed to doxorubicin and daunorubicin undergo morphological changes characteristic of programmed cell death, i.e., shrinkage, chromatin condensation, formation of apoptotic vesicles [138]. However, the intensity of these processes in cells treated with these antibiotics varies. At low micromolar concentrations, doxorubicin (≥5 µM) and daunorubicin (≥1 µM) increase the generation of reactive oxygen species, decrease intracellular reduced glutathione, induce an alteration in endothelial elasticity and cause a reorganization of the F-actin cytoskeleton [138].

Although the cited articles [99,137,138] mainly refer to the results of cell elasticity, which forms the basis of this discussion, other cellular parameters, including levels of nitric oxide, prostacyclin, calcium and reactive oxygen species, have also been examined and complement the results of force spectroscopy. Changes in cell elasticity parameters under the influence of drugs or cytokines do not directly reflect physiological or pathological changes in endothelial cells; however, they may indicate potential mechanisms initiated by these substances.

## 6. Toxicity of Nanostructures Versus Cell Elasticity

Nanostructures are widely studied in modern nanomedicine in the context of their use in drug delivery systems. Although the concept of using nanostructures to deliver drugs precisely to diseased tissue has been analyzed, it is already well-known that they may also impact healthy tissue which facilitates this transport. One objective of studying the effects of nanostructures on endothelial cells is to determine their toxicity. Apart from assessing cell viability in the presence of nanostructures using standard methods such as the spectrophotometric XTT test (3′-[1-[phenylamino-carbonyl]-3,4-tetrazolium]-bis(4-methoxy-6-nitro)-benzene-sulfonic acid hydrate) or the MTT test (3-(4,5-dimethylthiazol-2-yl)-2,5-diphenyltetrazolium bromide), it is also necessary to precisely determine changes in cell phenotype, including with nanostructure concentrations that do not affect viability. The effect of nanostructures on cells involves several aspects. These include the impact on the cell membrane, agglomeration on the membrane, transport into the cell, alterations in the cortical cytoskeleton and the emergence of stress fibers. It is also associated with the presence of nanomaterials or their agglomerates within cells, e.g., inside vesicles.

### 6.1. Impact of Carbon Nanotubes on Endothelial Cell Elasticity

EA.hy926 cells exposed to multi-walled carbon nanotubes at concentrations corresponding to 90% and 75% cell viability are stiffer than reference cells [20]. This is due to agglomeration of the carbon nanotubes on the cell membrane, agglomerates of the nanotubes inside cells, and polymerization of the actin fibers [20]. In addition to changes in cell elasticity caused by multi-walled carbon nanotubes, an increase in apical surface area and a decrease in cell roundness were observed, as well as an increase in the summit density (*Sd*) value [140]. Dong et al. [141] demonstrated that multi-walled carbon nanotubes increased the stiffness of human lung epithelial cells. Both articles [20,141] present evidence that higher stiffness of endothelial cells is correlated with an increase in reactive oxygen species (ROS) and changes in the cellular cytoskeleton.

### 6.2. Alteration in Endothelial Elasticity Induced by Metallic and Polymer Nanoparticles

In the case of cells exposed to silver nanoparticles [20,21,142], two-step response in the cell elasticity was observed [20]. At a concentration of SNPs corresponding to 90% cell viability, an increase in cell elasticity was detected. Whereas, cells treated with these nanoparticles at a concentration corresponding to 75% viability exhibited a higher elasticity parameter value than reference (untreated) cells. This is related to the polymerization and depolymerization processes of the actin cytoskeleton fibers, as well as the agglomeration of nanoparticles on or just below the cell membrane, which was also confirmed by scanning and transmission electron microscopy [20]. Surprisingly, the authors demonstrated that an increase in cell elasticity did not occur for higher passages of the EA.hy926 cell line, i.e., above passage 33 [21]. This effect may indicate a change in the cell phenotype that leads to resistance to silver nanoparticles in the EA.hy926 line [21]. Oliveira et al. [143] showed that non-toxic concentrations of metallic nanoparticles, including Fe_2_O_3_ and TiO_2_, also reduced the cell elasticity parameter related to the loss of stress fibers. Additionally, our previous work [144] showed that HUVECs exposed to gold nanoparticles stabilized with polyamidoamine dendrimers (AuNPs/PAMAMs) at non-toxic concentrations also reduced the cell elasticity parameter.

On the other hand, when exposed to SNPs at a non-toxic concentration (i.e., one for which no changes in viability were observed compared to untreated cells), endothelial cells are stiffer after short incubations of one and three hours [142]. However, after six hours of incubation, the elasticity values of these cells do not differ from those observed in the control group [142]. This dynamic change in elasticity for short incubation times is not associated with changes in cellular cytoskeletal actin. In the case of exposure to non-toxic concentrations of silver nanoparticles for short periods, a major role in the changes in elasticity of endothelial cells is probably played by the incorporation of nanoparticles into the glycocalyx, their endocytosis into the cell, and their accumulation in the cortical part of the cytoskeleton [142].

Interestingly, a biomechanical study reveals that elongated human aortic endothelial cells (HAECs) demonstrate greater cellular stress and stiffer membranes than cells with low aspect ratios, which proves that a clear relationship exists between morphology, mechanical phenotype and carboxylate polystyrene nanoparticle uptake [145]. Furthermore, when cocultured in the same chamber, endothelial cells elongated by high laminar shear endocytosed polystyrene nanoparticle considerably less than those that were not elongated in the chaotic, lower shear sections [145].

### 6.3. Effect of Dendrimers on Cell Elasticity

Additionally, in our previous article [146] we describe the influence of with polyamidoamine (PAMAM) dendrimers with ethylenediamine core of 2nd, 4th and 7th generation on endothelial cells. For cells treated with PAMAM dendrimers of the 2nd and 4th generation, a dose-dependent decrease in the elasticity parameter (measured with a sharp probe) was obtained compared to control (untreated) cells. This is related to the depolymerization of actin fibers and the induction of the early stage of apoptosis in cells [146]. For the spherical probe, a decrease in the cell elasticity parameter was also obtained; however, the effect was inversely proportional, i.e., the higher the concentration of PAMAM dendrimers, the higher the value of the cell elasticity parameter was. The authors of the article [146] indicate that it results from the accumulation and self-assembly of nanostructures in vesicles on the cell membrane or inside cells. Whereas in the case of cells treated with PAMAM dendrimers of the 7th generation at the concentration causing 50% cellular viability, an increase in the cell elasticity parameter was obtained, which is associated with the accumulation of nanostructures on the cell membrane. Table 1 illustrates a systematic summary of the effects of nanostructures on cells to assess changes in their mechanical properties. The summary includes information on the cell line, types of nanostructures, concentration studied, elasticity parameter value, and conclusions drawn from the conducted research.

Nanomaterials administered to endothelial cells in higher concentrations are toxic. Measurement of cell elasticity enables in-depth analysis of changes related to the accumulation of these nanostructures and remodeling of the cellular cytoskeleton. It should be noted that both the type of nanomaterial and its shape determine the type of impact on endothelial cells. Metallic nanoparticles can accumulate on or just below the cell membrane [146,147], thereby changing the cell elasticity. In the case of PAMAM dendrimers, the penetration process itself is crucial to changes in cell elasticity. Mecke et al. [148] demonstrated that for smaller cationic dendrimers with a diameter of less than 4 nm, dendrimer agglomeration occurs by building ledges surrounding the existing bilayer defects. Larger cationic dendrimers, with diameters ranging from 6 to 10 nm, interact with lipid bilayers to form highly swollen, fragmented bilayers and small dendrimer-filled lipid vesicles that enter the cell, leaving holes in the lipid bilayer/cell membrane [148,149,150]. The interaction model for the self-assembly of dendrimer-filled lipid vesicles hypothesized that dendrimers remove lipid molecules, creating membrane defects [148,149,150].

## 7. Finite Element Method in Endothelial Cell Mechanical Response

Analyzing the force response of living cells during AFM indentation is a complex issue in the study of the elasticity of soft materials. While standard models are commonly used for this analysis, there is a continuous search for new methods that can more accurately address this problem. One such promising method is the Finite Element Method (FEM) simulation. A key advantage of FEM is its ability to model the cell as a multi-layered structure. This chapter focuses on two major themes: modeling the cytoskeletal architecture and understanding viscoelastic and hyperelastic properties of endothelial cells.

### 7.1. Modeling of Cytoskeletal Architecture

Early FEM studies laid the groundwork for understanding how endothelial cells adapt to shear stress. Sato et al. [151] used 3D FEM to show that cells elongate and align with the direction of fluid flow, with actin filaments concentrating in central and polar regions. This alignment reflects known morphological adaptations. Yamaguchi et al. [152] further demonstrated that mechanical optimization drives spontaneous cell elongation and alignment under flow conditions. Ohashi et al. [153] combined atomic force microscopy (AFM) with FEM to quantify how shear stress alters local stiffness in bovine aortic endothelial cells. Their findings linked cytoskeletal remodeling directly to mechanical adaptation. Jean et al. [154] modeled the adhesion–cytoskeleton–nucleus pathway, showing how cytoskeletal tension affects nuclear deformation and mechanotransduction. Their model included key subcellular structures and tested two cytoskeletal configurations—discrete actin fibers and a homogeneous fiber network—both of which produced similar nuclear stretch values that matched experimental data. Ferko et al. [155] developed a multicomponent FEM model that incorporated focal adhesions and experimentally derived 3D topographies. Their work revealed stress amplification near adhesion sites and the nucleus, highlighting mechanical heterogeneity within the cell. Deguchi et al. [156] explored vertical intracellular force balance using a 3D FEM model, proposing that actin filaments generate vertical tension, microtubules bear compressive loads, and the nucleus serves as a central compression-bearing organelle. Wood et al. [157] introduced image-based FEM with discrete actin fibers, emphasizing that spatial arrangement—not just quantity—determines stiffness. Vargas-Pinto et al. [158] focused on human umbilical vein endothelial cells (HUVECs) and Schlemm’s canal cells, showing that the actin-rich cortex dominates AFM stiffness measurements. Their FEM simulations confirmed that sharp probes are especially sensitive to cortical mechanics. Jakka and Bursa [159] applied bendotensegrity modeling to simulate cytoskeletal flexure and prestrain, validating the roles of actin and microtubules in stiffness. Their model showed that removing actin filaments reduced stiffness by about 20–23%, while intermediate filaments and microtubules contributed to nonlinear responses under load. In a follow-up study [160], they extended this model to arterial wall loading, demonstrating that shear stress and axial stretch amplify nuclear strain. They found that actin filaments dominate stiffness under compression, while microtubules are more influential under shear stress. Yang et al. [161] constructed a detailed 3D FEM model to investigate how AFM probe geometry affects stiffness measurements. Their results showed that sharp probes detect localized stiff structures like stress fibers, while blunt probes provide more averaged mechanical responses. These findings underscore the importance of probe design in interpreting AFM data. Table 2 provides a comparative overview of key FEM-based studies on endothelial cytoskeletal architecture, summarizing modeling approaches, involved components, major findings, and each study’s unique contribution to the field.

FEM has also helped clarify how shear stress influences cell morphology, cytoskeletal organization, and gene expression [151,152,153]. Ohashi et al. [162] used structural optimization FEM to simulate cytoskeletal remodeling, confirming upstream stiffening and central stress fiber formation. Yamada et al. [163] employed image-based FEM to model 3D deformation under substrate stretch, highlighting the role of nuclear stiffness in shaping the cell.

### 7.2. Viscoelastic and Hyperelastic Modeling

Understanding how endothelial cells deform over time requires models that go beyond simple elasticity. Viscoelastic models capture time-dependent behaviors like stress relaxation, while hyperelastic models handle large, nonlinear deformations—especially relevant during inflammation, aging, or injury. Kang et al. [164] pioneered the use of the eight-chain hyperelastic model to simulate AFM indentation on pulmonary microvascular endothelial cells. Their model revealed depth-dependent stiffness and cytoskeletal changes under TNF-α treatment, outperforming traditional Hertzian models in accuracy. Wei et al. [165] introduced an inverse FEM approach using Prony series to extract viscoelastic parameters from AFM data. Their method proved robust across different cell types and indentation depths. Li et al. [166] developed a coupled FEM-agent-based model to simulate in-stent restenosis, integrating hyperelastic artery mechanics with endothelial regulation of smooth muscle proliferation. In another study [167], a multi-layered FEM model was used to simulate force responses during AFM indentation, with input parameters derived from experimental data. The model achieved less than 10% error compared to actual measurements. Wang et al. [33] combined AFM nanoindentation with viscohyperelastic FEM modeling to study how loading rate affects mechanical properties. They found that faster loading rates increased the measured elastic modulus, especially in short-term force peaks. Another study [168] used a hybrid FEM-AFM approach to identify mechanical biomarkers in renal tubule epithelial cells, both untreated and treated with an actin-disrupting drug. Peng et al. [169] used FEM and AFM to distinguish living from fixed HUVECs based on viscoelastic relaxation times, identifying 5 μm/s as the optimal rate for differentiation. Zhou et al. [170] refined FEM-based indentation models to correct errors in traditional assumptions, improving consistency across probe geometries and enhancing the accuracy of mechanical analysis.

## 8. Conclusions

This review article demonstrates how force spectroscopy can be used to study endothelial cells exposed to cytokines, hormones, drugs, and nanostructures. Because of their abundance in the human body, the elasticity parameters of these cells provide crucial information that contributes to physiological cellular processes.

A significant advantage of this method is its ability to study single cells and create elasticity parameter maps of any cell region. However, this method also poses many challenges such as selecting the appropriate measuring tip and analysis model. In this article, we present an elasticity model for drug testing, along with examples of its applications. Alterations in the elasticity parameters of cells resulting from drugs or cytokines cannot be directly translated into physiological changes or abnormal endothelial cells; however, they may indicate potential mechanisms triggered by these agents. When studying nanostructures, it is important to consider their agglomeration on the cell membrane since this factor can significantly impede or even prevent measurement.

Modeling the cytoskeleton has proven essential for capturing the structural complexity and mechanical diversity of endothelial cells. FEM simulations incorporating actin filaments, microtubules, and intermediate filaments can replicate localized stiffness variations and predict cellular responses to mechanical stimuli. Viscoelastic and hyperelastic models address the limitations of linear elasticity, offering deeper insights into how cells behave under physiological and pathological conditions.

Together, these approaches form a comprehensive framework for applying FEM in endothelial cell research. They broaden knowledge on cellular biomechanics and support the development of predictive models for vascular health, disease progression, and therapeutic strategies. Future work will benefit from integrating multiscale modeling, high-resolution imaging, and machine learning to further refine FEM applications in mechanobiology.

## Figures and Tables

**Table 1:** Summary of the effects of nanostructures on cells mechanical properties.

Cell Line and Nanostructures	Indentation Probe	Outcomes	Source
EA.hy926 cell line + Multi-walled carbon nanotubes (4 and 12 μg/mL)/Silver nanoparticles (3 and 3.6 μg/mL)/PAMAM dendrimers of 4th generation (0.4 and 0.8 μmol/L)	PFQNM-LC (Bruker); glass spheres (Nova Scan)	MWCNTs: ↑ *E* of 110% in parallel with an increase in ROS levels and alteration in actin cytoskeleton	[20]
		SNP: ↓ *E* of 43% (3 μg/mL), ↑ *E* of 112% (3.6 μg/mL) accompanied by thickening of actin fibers	
		PAMAM G4: ↓ *E* of 14% (0.8 μmol/L) and increase in apoptotic cells accompanied by an elevated level of ROS production	
BEAS-2B cell line + Multi-walled carbon nanotubes (24 µg/cm^2^)	Sharp probe	↑ *E* of 29% relative to control cells	[141]
EA.hy926 cell line + Silver nanoparticles (3, 3.6 and 16 μg/mL)	PFQNM-LC (Bruker); glass spheres (Nova scan)	Dose-dependent increase in *E* (of 14, 20 and 42% for selected SNPs concentrations, respectively) and polymerization of F-actin fibers in the central parts of the cells.	[21]
EA.hy926 cell line + Silver nanoparticles (1, 3, 3.6 and 16 μg/mL)	PFQNM-LC (Bruker)	Reduction in *E* of 16% (after 24 h incubation of 3 μg/mL SNPs); Increase in *E* (of 90 and 150% after one-hour incubation of 1 and 3 μg/mL of SNP) accompanied by the elevated ROS level	[142]
A-549 alveolar epithelial cells + Fe_2_O_3_ and TiO_2_ nanoparticles (10 μg/mL—no cytotoxic effect)	Spherical tip (Nova Scan)	Decreased cell stiffness compared to control cells of 28% and 24% for Fe_2_O_3_ and TiO_2,_ respectively	[143]
HUVEC cell line + PAMAM dendrimers of 2nd (1.08, 1.90 and 2.7 μmol/L), 4th (0.15, 0.45 and 0.95 μmol/L) and 7th (0.17, 0.35 and 0.65 μmol/L) generation	MLCT-SPH-spherical (Bruker); PFQNM-LC (Bruker)	PAMAM G2: dose-dependent decrease in E (of 19, 30 and 56% for selected concentrations, respectively); PAMAM G4: dose-dependent decrease in *E* (of 20, 59 and 67% for selected concentrations, respectively) PAMAM G7: ↓ *E* of 33% (0.35 μmol/L); ↑ *E* of 102% (0.65 μmol/L);	[146]
HUVEC + gold nanoparticles stabilized with PAMAM dendrimers (no cytotoxic: 0.5 and 0.7 μg/mL; and 1.1 μg/mL—80% cellular viability)	PFQNM-LC (Bruker)	↓ *E* of 34% for 0.5 μg/mL ↓ *E* of 24% for 0.7 μg/mL ↑ *E* of 28% for 1.1 μg/mL	[144]

*E* defined as elasticity parameter. Percentage changes in *E* relative to control (untreated) cells values.

**Table 2:** Summary of FEM approaches in endothelial cytoskeletal research.

Modeling Approach	Cytoskeletal Components	Main Results	Unique Contribution	Source
3D FEM with fluid–structure interaction	F-actin	Stress distribution correlates with F-actin localization	Early integration of AFM and FEM for shear stress analysis	[151]
CFD with dynamic cell modeling	Implicit cytoskeletal adaptation	Cell elongation and alignment reduce wall shear stress	Mechanically optimized morphogenesis simulation	[152]
AFM + FEM with axisymmetric model	F-actin	Shear stress increases stiffness and reorganizes cytoskeleton	Spatial stiffness mapping under flow	[153]
Axisymmetric FEM	AFs (discrete and continuum)	Cytoskeletal tension drives nuclear deformation	Mechanotransduction pathway modeling	[154]
Multicomponent FEM with FA mapping	Cytoplasm, nucleus, FAs	Stress amplification near FAs and nucleus	Quantified stress heterogeneity due to adhesion sites	[155]
3D FEM with vertical force balance	AFs, MTs	Actin compresses vertically; MTs resist compression	First model of vertical intracellular mechanics	[156]
3D FEM from confocal images	Actin stress fibers as truss elements	Spatial arrangement of fibers affects stiffness	Validated image-based FEM pipeline	[157]
FEM with two-layer cortex model	Actin cortex	Sharp AFM tips overestimate stiffness due to cortex	Demonstrated cortical dominance in AFM measurements	[158]
Hybrid FEM with bendotensegrity	AFs, MTs, IFs	AFs dominate stiffness; MTs contribute under tension	Realistic modeling of cytoskeletal flexure	[159]
3D FEM in arterial wall context	AFs, MTs, IFs	Shear stress and axial stretch amplify nuclear strain	Integration of cell mechanics with vascular loading	[160]
3D FEM with detailed cytoskeletal geometry	AFs, MTs, actin cortex	Probe geometry affects stiffness measurement	Spatially resolved stiffness mapping via FEM	[161]

Abbreviations: MTs—microtubules, AFs—actin filaments, IFs—intermediate filaments.

## Data Availability

No new data were created or analyzed in this study. Data sharing is not applicable to this article.

## Abbreviations

The following abbreviations are used in this manuscript:

AFM	Atomic force microscopy
AFS	Atomic force spectroscopy
ECs	Endothelial cells
HMEC	Human microvascular endothelial cells
HUVEC	Human umbilical vein endothelial cells
SNP	Silver nanoparticles
MWCNT	Multi-walled carbon nanotubes
PAMAM	Polyamidoamine dendrimers
CAT	Cationic amino acid transporters
NO	Nitric oxide
eNOs	Endothelial nitric oxide synthase
PGI_2_	Prostacyclin
AA	Arachidonic acid
PKA	Protein kinase A
PLA_2_	Phospholipase A2
PGG_2_	Prostaglandin G2
PGH_2_	Prostaglandin H2
COX	Cyclooxygenase
ROS	Reactive oxygen species
PAF	Platelet-activating factor
ET-1	Endothelin-1
ENaC	Endothelial sodium channels
TNF-α	Tumor necrosis factor—α
Sd	Summit density
DOX	Doxorubicin
DNR	Daunorubicin
FEM	Finite Element Method
AFs	Actin filaments
MTs	Microtubules
IFs	Intermediate filaments
